# Comparative risk assessment of school food environment policies and childhood diets, childhood obesity, and future cardiometabolic mortality in the United States

**DOI:** 10.1371/journal.pone.0200378

**Published:** 2018-07-06

**Authors:** Katherine L. Rosettie, Renata Micha, Frederick Cudhea, Jose L. Peñalvo, Martin O’Flaherty, Jonathan Pearson-Stuttard, Christina D. Economos, Laurie P. Whitsel, Dariush Mozaffarian

**Affiliations:** 1 Friedman School of Nutrition Science & Policy, Tufts University, Boston, Massachusetts, United States of America; 2 Department of Public Health and Policy, University of Liverpool, Liverpool, United Kingdom; 3 School of Public Health, Imperial College London, London, United Kingdom; 4 American Heart Association, Arlington, Virginia, United States of America; University of Adelaide School of Medicine, AUSTRALIA

## Abstract

**Background:**

Promising school policies to improve children’s diets include providing fresh fruits and vegetables (F&V) and competitive food restrictions on sugar-sweetened beverages (SSBs), yet the impact of national implementation of these policies in US schools on cardiometabolic disease (CMD) risk factors and outcomes is not known. Our objective was to estimate the impact of national implementation of F&V provision and SSB restriction in US elementary, middle, and high schools on dietary intake and body mass index (BMI) in children and future CMD mortality.

**Methods:**

We used comparative risk assessment (CRA) frameworks to model the impacts of these policies with input parameters from nationally representative surveys, randomized-controlled trials, and systematic reviews and meta-analyses. For children ages 5–18 years, this incorporated national data on current dietary intakes and BMI, impacts of these policies on diet, and estimated effects of dietary changes on BMI. In adults ages 25 and older, we further incorporated the sustainability of dietary changes to adulthood, effects of dietary changes on CMD, and national CMD death statistics, modeling effects if these policies had been in place when current US adults were children. Uncertainty across inputs was incorporated using 1000 Monte Carlo simulations.

**Results:**

National F&V provision would increase daily fruit intake in children by as much as 25.0% (95% uncertainty interval (UI): 15.4, 37.7%), and would have small effects on vegetable intake. SSB restriction would decrease daily SSB intake by as much as 26.5% (95% UI: 6.4, 46.4%), and reduce BMI by as much as 0.7% (95% UI: 0.2, 1.2%). If F&V provision and SSB restriction were nationally implemented, an estimated 22,383 CMD deaths/year (95% UI: 18735, 25930) would be averted.

**Conclusion:**

National school F&V provision and SSB restriction policies implemented in elementary, middle, and high schools could improve diet and BMI in children and reduce CMD mortality later in life.

## Introduction

Diets of American youth are suboptimal, contributing to obesity in childhood and type 2 diabetes and cardiovascular disease (CVD) later in life [1–3]. According to the Institute of Medicine, schools are an essential setting for policies aimed at improving the diets of children and adolescents (hereafter referred to as children) [4]. Children consume over one-third of their daily food in school [4]; and childhood represents a crucial formative period given that long-term dietary preferences form early in life and that both dietary habits and obesity tend to track into adulthood [1–3,5].

Two promising school food environment policies include provision of fresh fruits and vegetables (F&V) and competitive food restrictions on sugar-sweetened beverages (SSBs) [6–9]. Increasing F&V and reducing SSBs could be beneficial to health in childhood and later in life, especially related to adiposity and cardiometabolic disease (CMD) [10,11]. F&V provision in schools involves the distribution of free or subsidized fresh fruits and vegetables to students, often as snacks offered outside of school meals [12–14]. SSB restriction includes limiting the availability, portion sizes, or sales of SSBs in schools [15–17]. Based on the promise of such policies, in 2008 the Fresh Fruit and Vegetable Program (FFVP) was expanded nationally for elementary schools with the highest low-income enrollments to provide free fresh F&V to students outside usual school meals [18]. In 2010 the Healthy, Hunger-Free Kids Act (HHFKA) introduced Smart Snack Standards in schools receiving federal meal funding. Among other standards including age-appropriate portion sizes, the HHFKA focused on restricting SSBs in public schools, with full implementation planned for 2016 [19].

However, the impact of implementing F&V provision and SSB restriction in elementary, middle, and high schools at a national scale on dietary intake and BMI in children is unknown. In addition, how the dietary effects of such policies would track into adulthood and potentially influence CMD mortality is not established. Understanding these potential effects is crucial to estimate the benefits of existing programs, including their expansion, and also elucidate the possible harms from elimination of such programs based on uncertain current federal priorities.

To address these gaps in knowledge, we used a comparative risk assessment (CRA) framework to estimate the quantitative impact of national implementation of F&V provision and SSB restriction in elementary, middle, and high schools in the US on dietary intake and BMI in children age 5–18 years and future CMD mortality in adults ages 25 and older. This investigation was performed as part of the Food-PRICE (Policy Review and Intervention Cost-Effectiveness) Project (www.food-price.org).

## Methods

### Current dietary intakes, demographics, and BMI in children

Data on mean dietary intakes, demographics, and BMI and corresponding uncertainty in children ages 5–18 years were obtained from the two most recent cycles of the National Health and Nutrition Examination Survey (NHANES) in 2009–10 and 2011–12 (N = 4,165), preceding widespread implementation of the Smart Snacks Standards or FFVP [20,21]. The FFVP was implemented in 2008 in the lowest income elementary schools, but not other elementary schools or middle or high schools, so dietary changes in this subset of schools may already be partially captured in NHANES 2009–11. The HHFK Act was passed in 2010, but not fully implemented until 2016, so it is unlikely that its effects would greatly alter our baseline SSB intake estimates from NHANES.

The NHANES 24-hour recall protocol and data collection methods are thoroughly documented elsewhere [22]. We used survey weights that accounted for the sample design and probabilities of selection to obtain nationally representative data for children. Data from two 24-hour recalls were averaged for each participant and dietary exposure, further accounting for within-person variation in determining distributions. The US Department of Agriculture Food Patterns Equivalents Database for years 2009–10 and 2011–12 was used to disaggregate multi-component foods into specific food groups, including fruits, vegetables, and SSBs [23,24]. The definitions used for these dietary exposures have been recently published [25]. NHANES demographic data were used to stratify dietary intakes by age (5–10 years [elementary school], 11–14 years [middle school], 15–18 years [high school]), race/ethnicity (non-Hispanic white, non-Hispanic black, Hispanic, other), and sex. BMI was obtained and similarly stratified by age, race/ethnicity, and sex, as directly measured in NHANES by trained personnel using standardized methods.

### Effects of F&V provision and SSB restriction on dietary intakes in children

The effects of school policies for F&V provision and SSB restriction on the consumption of fruits, vegetables, and SSBs in children were obtained from a meta-analysis of intervention studies, including 6 randomized trials, 8 quasi-experimental studies, and 1 regression discontinuity study of F&V provision programs, and 3 quasi-experimental trials of SSB restriction programs [6]. We focused on the effect sizes of each intervention on total food consumption, not only in-school intakes, using 24-hour recalls and food frequency questionnaires to account for any compensatory dietary changes outside of school. The identified trials were generally 1–2 years in duration, and therefore reflected the 1–2 year effects of these policies.

### Etiologic effects of dietary changes on BMI in children

We conservatively assumed no effects of F&V consumption on childhood BMI in our main analysis, based on insufficient evidence linking F&V intake to BMI in childhood [26,27]. In sensitivity analysis, we used pooled estimates from 3 large cohort studies in adults to estimate the relationship between changes in fruit and vegetable intake and changes in BMI in children [28]. For SSBs, the change in BMI per serving change in daily SSB intake was obtained from a randomized controlled trial in children [29], after confirming similar findings in a second randomized trial [30] as well as long-term observational studies (**S1 File, S1 Table**) [31].

### Current dietary intakes, demographics, and BMI in adults

Data on mean dietary intakes, demographics, and BMI and corresponding uncertainty in adults ages 25 and older were obtained from NHANES 2009–2010 and 2011–2012 (N = 8,510) using similar methods as described for children [20,21]. Dietary intakes and BMI were stratified by age (25–34, 35–44, 45–54, 55–64, 65–74, 75+ years), race/ethnicity, and sex.

### Effects of childhood dietary changes sustained into adulthood

We estimated the effects of these policies on long-term changes in dietary intake from a systematic review of cohort studies that correlated within-individual dietary intakes from childhood to adulthood [3]. The average correlation was approximately 0.35 or higher for most dietary habits [3]. Within-individual correlations of dietary intakes were comparable over various durations of follow-up, and for different nutrients and food categories. Data on the sustained absolute differences in these dietary intakes were not reported. In our main analysis, we used these findings to assume that 35% of the dietary changes associated with school policies in childhood would be sustained into adulthood; and varied this assumption from 25% to 50% in sensitivity analyses.

### Etiologic effects of dietary changes on disease outcomes

The associations of dietary changes with disease-specific mortality in adults were obtained as previously described from a systematic review of meta-analyses of prospective cohorts and randomized trials [32]. This included effect estimates for consumption of fruits, vegetables, and SSBs in relation to CHD, stroke, and type 2 diabetes (**S2 File**). Data on disease-specific deaths in US adults by age, sex, and race/ethnicity were obtained from the National Center for Health Statistics for 2012, including deaths from coronary heart disease, ischemic stroke, hemorrhagic stroke, unidentified and other non-ischemic/hemorrhagic stroke, and diabetes mellitus [33].

### Data analysis

A CRA modeling framework was utilized as previously described [32], including probabilistic sensitivity analysis using 1000 Monte Carlo simulations to jointly incorporate the uncertainty of multiple inputs including baseline dietary intakes and BMI, effects of the school policies on dietary changes, effects of dietary changes on BMI in childhood, and effects of dietary changes on CMD mortality later in life.

#### Dietary intakes and BMI in children

We modeled the short-term (1–2 year) impact of national implementation of school-based F&V provision and SSB restriction on habitual dietary intakes in US children age 5–18 years. To assess 1–2 year changes in adiposity, we incorporated the estimated effect of changes in habitual SSB intake on BMI [29]. We conservatively assumed that changes in F&V had no effect on BMI; we varied this assumption in a sensitivity analysis.

#### Cardiometabolic mortality in adults

Because forward projection over 70+ years would require challenging assumptions about long-term trends in prevalent dietary habits, adiposity, and CMD mortality, we estimated the impact of these school policies on CMD in adults under the paradigm of how current CMD rates might differ if such policies were in place when all current adults had been schoolchildren. We estimated the long-term effects of F&V provision and SSB restriction on CMD mortality using the CRA model in adults with the following inputs: (1) baseline adult dietary intakes and BMI by age, race/ethnicity, and sex; (2) effects of F&V provision and SSB restriction on childhood diets; (3) extent of childhood dietary changes sustained into adulthood; (4) etiologic effects of dietary changes in adulthood on disease outcomes; and (5) baseline mortality estimates. Because the usual duration of the school policy intervention trials was only 1–2 years, we recognized that the findings from these studies might underestimate the effects of extended national policies throughout elementary, middle, and high school. Yet, we also recognized that assuming fully additive effects every 1–2 years might be overly optimistic. We therefore utilized an intermediate assumption that such policies, implemented across all years of schooling, would have one effect during elementary school (+0.27 servings/d fruit, +0.04 servings/d vegetables, -0.27 servings/d SSBs), one effect during middle school, and one effect during high school. We then modeled that 35% of such changes would be sustained into adulthood (sensitivity range: 25%, 50%). For each age, sex, and race/ethnicity stratum, we estimated the proportion of disease-specific deaths averted by each policy by computing the potential impact fraction (PIF). The joint impact of dietary changes associated with these policies were estimated by calculating the joint PIF. Additional details on the comparative risk assessment methodology can be found in the technical appendix **(S2 File),** and additional descriptions on each of the model inputs can be found in the supplementary material **(S2 Table)**. Model inputs were prepared using Stata version 14 [34], and analyses were conducted using R version 3.1.0 [35].

## Results

### F&V provision and SSB restriction policies and diet and BMI in children

**Fig 1:**
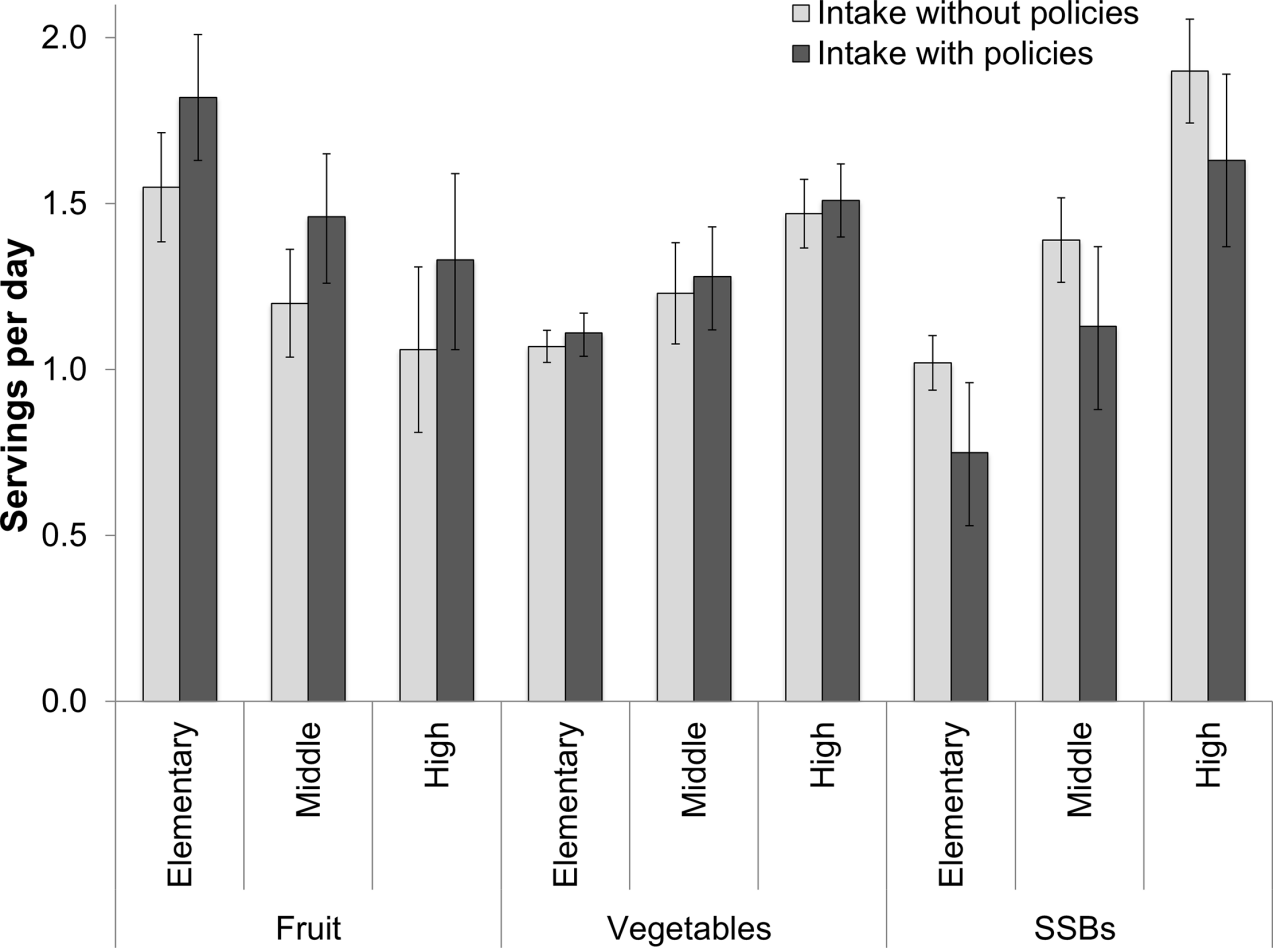
Current and estimated changes in dietary intakes associated with national school policies on F&V provision and SSB restriction among US children by age. Current intakes are based on NHANES 2009–10 and 2011–12 (N = 4,165 children age 5–18 years), where bars represent the mean and error bars, the 95% confidence intervals. Estimates for dietary intake with policies are based on a comparative risk assessment framework incorporating policy effects from intervention studies, where bars represent the median values from 1,000 Monte Carlo simulations and error bars, the 95% uncertainty intervals.

We estimated that F&V provision in schools would increase habitual fruit intake by 17.1% in elementary school students (95% uncertainty interval (UI): 10.8, 23.6%), 22.2% in middle school students (95% UI: 14.1, 31.5%), and 25.0% in high school students (95% UI: 15.4, 37.7%) over 1–2 years (**Table 1; Fig 1**). This policy was estimated to have a much smaller effect on vegetable intake in children. SSB restriction in schools would change habitual SSB intake by an estimated -26.5% (95% UI: 6.4, 46.4), 19.2% (95% UI: 4.5, 34.5%), and 14.5% (95% UI: 3.4, 25.2%) among elementary, middle, and high school students, respectively. By child gender, we estimated that F&V provision would lead to a 21.3% (95% UI: 13.5, 30.1%) and a 19.4% (95% UI: 12.2, 27.2%) increase in fruit intake in males and females, respectively, while vegetable intake would increase by 3–4% in males and females (**S3 Table**). SSB restriction would lead to a slightly larger reduction in SSB intake in males than females (20.5% [95% UI: 5.3, 36.1%] in males versus 18.5% [95% UI: 4.5, 32.5%] in females). Stratified by race, we estimated increases in fruit intake would range from 18.1% (95% UI: 11.4, 25.1%) among Hispanics to 27.4% (95% UI: 17.3, 37.6%) among non-Hispanic blacks to, while decreases in SSB intake would be as large as 26.8% (95%UI: 6.3, 48.5%) among children in the other race category (**S4 Table**).

**Table 1:** Select model inputs and estimated changes in child diet and child BMI associated with F&V provision and SSB restriction in US schools^1^.

Dietary exposure	Child school level	Baseline dietary intake, mean servings/day (SE)^2^	Dietary change associated with F&V provision and SSB restriction (servings/day)		Baseline BMI, mean kg/m^2^ (SE)^5^	BMI change associated with SSB restriction^5^	
			Mean (SE)^3^	% (95% UI)^4^		Median (95% UI)	% (95% UI)
**Fruits** (80g/svg)							
	Elementary school	1.6 (0.1)	0.27 (0.05)	17.1 (10.8, 23.6)	17.8 (0.1)	NA	NA
	Middle school	1.2 (0.1)	0.27 (0.05)	22.2 (14.1, 31.5)	22.1 (0.2)	NA	NA
	High school	1.1 (0.1)	0.27 (0.05)	25.0 (15.4, 37.7)	24.6 (0.2)	NA	NA
**Vegetables** (80g/svg)							
	Elementary school	1.1 (0.03)	0.04 (0.02)	4.1 (0.4, 7.8)	17.8 (0.1)	NA	NA
	Middle school	1.2 (0.1)	0.04 (0.02)	3.6 (0.5, 6.8)	22.1 (0.2)	NA	NA
	High school	1.5 (0.1)	0.04 (0.02)	3.0 (0.3, 5.7)	24.6 (0.2)	NA	NA
**SSBs** (8oz/svg)							
	Elementary school	1.0 (0.04)	-0.27 (0.10)	-26.5 (-46.4, -6.4)	17.8 (0.1)	-0.12 (-0.21, -0.03)	-0.7 (-1.2, -0.2)
	Middle school	1.4 (0.1)	-0.27 (0.10)	-19.2 (-34.5, -4.5)	22.1 (0.2)	-0.12 (-0.21, -0.03)	-0.5 (-1.0, -0.2)
	High school	1.9 (0.1)	-0.27 (0.10)	-14.5 (-25.2, -3.4)	24.6 (0.2)	-0.12 (-0.21, -0.03)	-0.5 (-0.8, -0.1)

Abbreviations: SSBs, sugar-sweetened beverage; F&V, fruit and vegetable; SE, standard error; UI, uncertainty interval; BMI, body mass index; NA, not applicable.

^1^ Outcomes were modeled assuming all US children in elementary, middle, and high school would be subject to F&V provision and SSB restriction. Students in private schools were not excluded because they constitute less than 10% of all US students and because the majority of private schools (tax-exempt, non-profit) could be subject to these policies. Outcomes are estimated using inputs for the effects of short-term (1–2 years) school food environment interventions on diet; no assumptions are made about the potential effects of longer-term policies on child diet and BMI.

^2^ Included the two most recent cycles of the National Health and Nutrition Examination Survey (NHANES; 2009–10 and 2011–12); N = 4,165 children ages 5–18 yrs. We accounted for survey design and sample weights, and averaged data from two nonconsecutive 24-hour dietary recalls. Energy-adjusted dietary intakes were calculated using the residual method.

^3^ Estimates of the impact of F&V provision and SSB restriction on absolute change (mean, SE) in dietary intake were obtained from a meta-analysis including 18 school food environment intervention studies. Studies included in this meta-analysis include interventions with durations ranging from approximately 1–2 years; therefore, estimated results for diet and BMI reflect the effects of these short-term interventions on absolute change in intake.

^4^ Point estimates and 95% uncertainty intervals for the percent change in dietary intake associated with F&V provision and SSB restriction were obtained from a probabilistic sensitivity analysis sampling from the distribution of dietary intakes of fruits, vegetables, and SSBs (mean, SE) obtained NHANES and the estimated effect of these policies on diet from a meta-analysis of school food environment intervention studies. The percent change is the median estimate from 1000 Monte Carlo simulations and the 95% uncertainty intervals are the 2.5^th^ and 97^th^ percentiles of the percent change.

^5^ Baseline (without policy) BMI data were obtained from the two most recent cycles of NHANES (2009–10 and 2011–12); N = 4723. The effect of changes in SSB intake on BMI was derived from a randomized controlled trial. Point estimates and 95% uncertainty intervals for the absolute and percent change in BMI were derived from probabilistic sensitivity analysis sampling from the distribution of baseline BMI (mean, SE) from NHANES, the estimated effects of SSB restrictions on SSB intake from a meta-analysis of school food environment interventions, and estimates for the relationships between changes in SSB intake and BMI from an RCT. The point estimates (median and percent change) are the median estimates from 1000 Monte Carlo simulations and the 95% uncertainty intervals are the 2.5^th^ and 97^th^ percentiles of the absolute and percent change in BMI. Due to insufficient evidence linking fruit and vegetable intake to BMI in childhood, we conservatively assumed no effects of F&V changes on childhood BMI in our main analysis.

A national school policy to restrict SSBs was estimated to change childhood BMI by -0.7% (95% UI: -1.2, -0.2%), -0.5% (95% UI: -1.0, -0.2%), and -0.5% (95% UI: -0.8, -0.1%) over 1–2 years for children in elementary, middle, and high school, respectively (**Table 1**). In sensitivity analyses including potential effects of F&V on BMI, a national school policy for F&V provision would lead to an approximate 0.1% additional reduction in BMI, largely related to increased fruit intake (**S5 Table**).

### Estimated cardiometabolic deaths averted among adults

**Fig 2:**
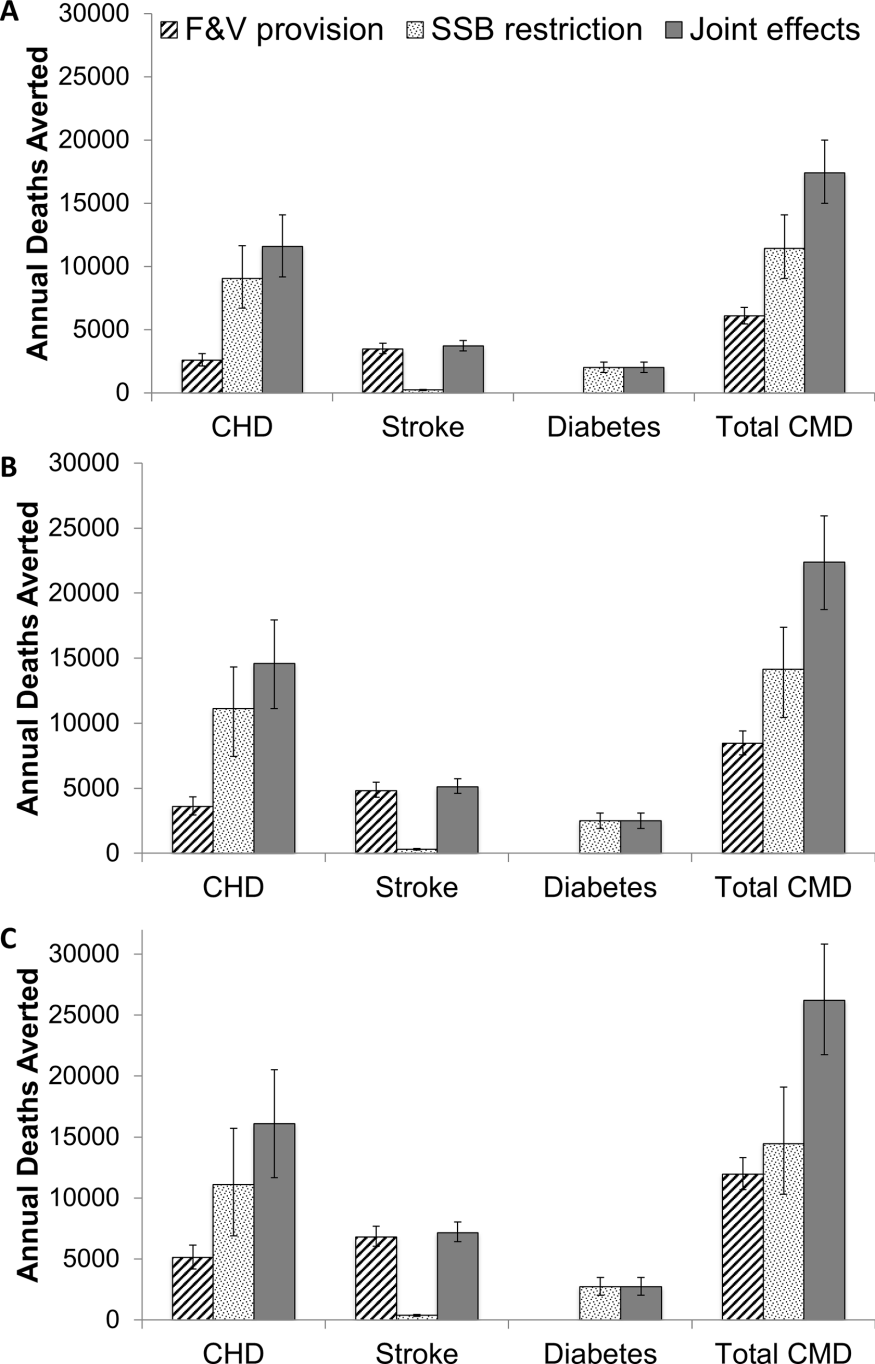
Estimated annual cardiometabolic deaths averted from implementation of national school policies on F&V provision and SSB restriction, separately and jointly, in US elementary, middle, and high schools. Bars represent the median values from 1,000 Monte Carlo simulations in a comparative risk assessment framework; and error bars, the 95% uncertainty intervals. Health effects are estimated for the current US adult population if exposed to these school environment policies during their childhood in elementary, middle, and high school. Panel (**A)** assumes that 25% of the dietary changes achieved in childhood are sustained into adulthood; panel (**B)**, that 35% of dietary changes are sustained; and panel (**C)** that 50% of dietary changes are sustained.

If such school policies had been implemented when current US adults were children, F&V provision would increase fruit intake in adults by 19.1% (95% UI: 12.3, 25.7), vegetable intake by 2.2% (95% UI: 0.5, 4.0%), and decrease SSB intake by 24.2% (95% UI: 5.1, 43.4%) (**Table 2**). F&V provision and SSB restriction were estimated to jointly prevent 22,383 CMD deaths/year (95% UI: 18735, 25930), or 3.2% of total annual CMD deaths (95% UI: 2.7, 3.7%) (**Table 3**). Among individual dietary targets, reductions in SSBs were estimated to have the greatest impact, with 14,132 deaths averted/year (95% UI: 10453, 17365) (**Table 3, Fig 2**). Increases in fruit intake associated with F&V provision were estimated to avert 7,457 CMD deaths per year (95% UI: 6615, 8428), while changes in vegetable intake had a small effect (983 CMD deaths averted/year; 95% UI: 790, 1217). For subtypes of CMD, CHD mortality would be most reduced by SSB restriction (3.0% reduction; 95% UI: 2.0, 3.9%), while stroke mortality would be most reduced by provision of fruit (3.4% reduction; 95% UI: 3.0, 3.8%). Diabetes mortality was only influenced by SSB restriction, with a 3.7% annual mortality reduction (95% UI: 2.8, 4.5%).

**Table 2:** Select model inputs for modeling CMD outcomes in adults.

	Baseline dietary intake, mean servings/day (SE)^1^	Dietary change associated with F&V provision and SSB restriction, servings/day^2^		Risk ratios for CMD mortality per serving/day, mean (95% CI)^4^		
		Mean (SE)	% (95% UI)^3^	CHD	Stroke	Diabetes
**Fruits** (80g/svg)	1.5 (0.03)	0.28 (0.05)	19.1 (12.3, 25.7)	0.94 (0.91, 0.98)	0.88 (0.83, 0.93)	NA^5^
**Vegetables** (80g/svg)	2.3 (0.04)	0.05 (0.02)	2.2 (0.5, 4.0)	0.95 (0.93, 0.98)	0.83 (0.74, 0.93)	NA^5^
**SSBs** (8oz/svg)	1.1 (0.04)	-0.28 (0.11)	-24.2 (-43.4, -5.1)	1.21 (1.13, 1.30)	NA^5^	1.22 (1.09, 1.36)

Abbreviations: SSBs, sugar-sweetened beverage; F&V, fruit and vegetable; SE, standard error; UI, uncertainty interval; CI, confidence interval; NA, not applicable; CHD, coronary heart disease.

^1^ Included the two most recent cycles of the National Health and Nutrition Examination Survey (NHANES; 2009–10 and 2011–12); N = 8,516 adults ages 25 and older We accounted for survey design and sample weights, and combined data from two nonconsecutive 24-hour dietary recalls.

^2^ Estimates of the impact of F&V provision and SSB restriction on absolute change (mean, SE) in dietary intake were obtained from a meta-analysis including 18 school food environment intervention studies. Studies included in this meta-analysis include interventions with durations ranging from approximately 1–2 years. Given the short duration of these interventions, we estimated the potential impact of these policies on diet in adults if they had been in place during their childhood by modeling the additive effects of F&V provision and SSB restriction across elementary, middle, and high school. Our model also assumed that 35% of dietary changes in childhood would be sustained into adulthood if the current US adult population had been exposed to these school environment policies during childhood, based on evidence in a systematic review on within-individual correlations of dietary habits in childhood and adulthood.

^3^ Point estimates and 95% uncertainty intervals for the percent change in dietary intake associated with F&V provision and SSB restriction were obtained from a probabilistic sensitivity analysis sampling from the distribution of dietary intakes of fruits, vegetables, and SSBs (mean, SE) from NHANES and the estimated effect of these policies on diet from a meta-analysis of school food environment interventions (accounting for additive effects and 35% sustainability into adulthood). The percent change is the median estimate from 1000 Monte Carlo simulations and the 95% uncertainty intervals are the 2.5^th^ and 97^th^ percentiles of the percent change

^4^ Risk ratio estimates correspond to a one serving increase in dietary intake for all adults ages 55–64. Risk ratio estimates were obtained from meta-analyses of cohort studies, with updated risk ratios and 95% CIs from our work in the 2010 Global Burden of Diseases Study. Estimates were obtained by age group 25–34 to 75+, with additional stratifications by sex and race, when appropriate.

^5^ We conservatively assumed there is no etiologic effect given insufficient evidence.

**Table 3:** Estimated cardiometabolic deaths averted associated with F&V provision and SSB restriction in US schools^1^.

		25% of dietary change sustained		35% of dietary change sustained		50% of dietary change sustained	
	Current deaths, 2012^2^	No. of deaths averted per year (95% UI)	Proportion (%) of deaths averted per year (95% UI)	No. of deaths averted per year (95% UI)	Proportion (%) of deaths averted per year (95% UI)	No. deaths averted per year (95% UI)	Proportion (%) deaths averted per year (95% UI)
*Fruit*							
CHD	371266	2262 (1810, 2779)	0.6 (0.5, 0.7)	3154 (2525, 3874)	0.8 (0.7, 1.0)	4479 (3587, 5499)	1.2 (1.0, 1.5)
Stroke	128294	3105 (2732, 3534)	2.4 (2.1, 2.8)	4309 (3793, 4903)	3.4 (3.0, 3.8)	6074 (5351, 6913)	4.7 (4.2, 5.4)
Total CVD	634394	5362 (4756, 6063)	0.8 (0.7, 1.0)	7457 (6615, 8428)	1.2 (1.0, 1.3)	10547 (9359, 11910)	1.5 (1.3, 1.7)
*Vegetables*							
CHD	371266	316 (212, 437)	0.1 (0.1, 0.1)	443 (297, 612)	0.1 (0.1, 0.2)	632 (423, 874)	0.2 (0.1, 0.2)
Stroke	128294	385 (288, 510)	0.3 (0.2, 0.4)	539 (403, 714)	0.4 (0.3, 0.6)	768 (575, 1018)	0.6 (0.4, 0.8)
Total CVD	634394	702 (565, 870)	0.1 (0.1, 0.1)	983 (790, 1217)	0.2 (0.1, 0.2)	1402 (1128, 1736)	0.2 (0.2, 0.2)
*SSBs*							
CHD	371266	9046 (6708, 11624)	2.4 (1.8, 3.1)	11121 (7463, 14328)	3.0 (2.0, 3.9)	11111 (6905, 15702)	3.0 (1.9, 4.2)
Stroke	128294	227 (187, 270)	0.2 (0.1, 0.2)	306 (253, 368)	0.2 (0.2, 0.3)	372 (295, 451)	0.3 (0.2, 0.4)
Diabetes	67914	2013 (1605, 2443)	3.0 (2.4, 3.6)	2514 (1904, 3088)	3.7 (2.8, 4.5)	2730 (2024, 3488)	4.0 (3.0, 5.1)
Total CMD	702308	11424 (9057, 14066)	1.6 (1.3, 2.0)	14132 (10453, 17365)	2.0 (1.5, 2.5)	14467 (10297, 19110)	2.1 (1.5, 2.7)
*Joint effects*^3^							
CHD	371266	11561 (9158, 14074)	3.1 (2.5, 3.8)	14601 (11128, 17949)	3.9 (3.0, 4.8)	16110 (11674, 20525)	4.3 (3.1, 5.5)
Stroke	128294	3696 (3321, 4143)	2.9 (2.6, 3.2)	5121 (4598, 5739)	4.0 (3.6, 4.5)	7155 (6415, 8044)	5.6 (5.0, 6.3)
Diabetes	67914	2013 (1605, 2443)	3.0 (2.4, 3.6)	2514 (1904, 3088)	3.7 (2.8, 4.5)	2730 (2024, 3488)	4.0 (3.0, 5.1)
Total CMD	702308	17390 (14978, 19975)	2.5 (2.1, 2.8)	22383 (18735, 25930)	3.2 (2.7, 3.7)	26210 (21741, 30818)	3.7 (3.1, 4.4)

Abbreviations: SSBs, sugar-sweetened beverage; F&V, fruit and vegetable; UI, uncertainty interval; CMD, cardiometabolic disease; HD, heart disease; CVD, cardiovascular disease; CHD, coronary heart disease

^1^ Estimated using a comparative risk assessment model. We assumed that the current US adult population had been exposed to these school environment policies during childhood from elementary school through high school. Our model also assumed that effects of school food environment interventions on diet would be additive across levels of schooling (elementary, middle, and high school) if current US adults had been exposed to these policies throughout childhood. In addition, we modeled 35% of dietary changes in childhood being sustained into adulthood, based on evidence in a systematic review on within-individual correlations of dietary habits in childhood and adulthood. In sensitivity analyses, we considered smaller (25%) and larger (50%) sustained changes in adulthood. The effects of dietary changes in adulthood on CMD mortality were obtained from meta-analyses of cohort studies, with updated relative risks and 95% CIs from our work in the 2010 Global Burden of Diseases Study. Point estimates and 95% uncertainty intervals were derived from probabilistic sensitivity analysis using 1000 Monte Carlo simulations.

^2^ Data on current CMD (ischemic heart disease, hypertensive heart disease, ischemic stroke, hemorrhagic and other non-ischemic stroke, and diabetes mellitus) deaths by age and sex derived from the National Center of Health Statistics, including a total of 702,308 CMD deaths in 2012.

^*3*^ Based on multiplicative attributable fractions for joint effects of changes in fruits, vegetables, and SSBs combined.

In sensitivity analyses assuming that either 25% or 50% of the dietary changes associated with these school policies were sustained into adulthood, an estimated 17,390 (95% UI: 14978, 19975) and 26,210 (95% UI: 21741, 30818) CMD deaths would be averted per year, respectively (**Table 3, Fig 2)**.

## Discussion

Our findings, based on nationally representative data and estimates from intervention studies of school policies and dietary habits, provide estimates of the potential impact of national implementation of F&V provision and SSB restriction policies on diet and BMI in children and CMD mortality in adults. To our knowledge, this is the first analysis to quantify these potential effects. Our results suggest that these policies would produce moderate but meaningful changes in diet over 1–2 years, with small corresponding changes in childhood BMI. If implemented across elementary, middle, and high schools, our results further suggest that 22,383 CMD deaths/year, or about 3% of the total national burden, would be averted in adults.

### Plausibility and coherence of findings

Relative effects by age and race reflect current intake patterns in US schoolchildren. For example, fruit intake is lower in high school than elementary school students, while SSB intake is nearly twice as high in high school compared to elementary school students [20,21]. The small effect on habitual vegetable consumption of typical school policies to provide fresh F&V is consistent with prior analyses [36, 37]. This may reflect both a stronger student preference for fruit as well as the relative ease and practicality for schools of serving fruit outside of usual school meals, compared with vegetables.

We identified a modest effect on childhood BMI of school restrictions on SSBs over 1–2 years, consistent with prior empiric experiences [38–40]. By 2009–12, many US schools had already reduced in-school SSB intakes due to educational or other policies, which would limit the estimated magnitude of implementing national school SSB restrictions. In addition, the majority of SSB consumption by children occurs outside of school, especially at home and also at restaurants and retail outlets [41]. Furthermore, the observed intervention effects incorporate the potential for partial compensation by increased SSB consumption outside of school. For all these reasons, our school-based intervention would be expected to have a modest impact on habitual SSB intake. However, even a small change in childhood BMI is relevant across the US population, and translation of even a portion of this lower SSB intake into adulthood diets led to substantial numbers of fewer CHD and diabetes deaths.

Very few studies have evaluated the long-term effects of childhood interventions on CMD outcomes later in life. Two cohorts have examined the long-term associations of dietary habits in childhood with cardiovascular risk and outcomes in young adulthood [42–45]. For example, the Cardiovascular Risk in Young Finns Study followed children ages 9–18 years for up to 27 years and found that children with higher vegetable consumption had a 14% lower risk of metabolic syndrome during adulthood, especially due to lower blood pressure and trigylcerides. The association persisted after adjustment for adulthood vegetable consumption, suggesting benefits of lifelong dietary habits (not captured in our analysis) [42]. Similar findings were reported in this cohort for childhood F&V consumption or generally healthy childhood diets combined with other healthy behaviors in relation to arterial pulse wave velocity and stiffness in adulthood [43,44]. A second long-term cohort following British children from 1937–39 found higher childhood intake of vegetables, but not fruit or other foods, was linked to lower risk of subsequent stroke [45]. Our investigation builds upon and extends these findings by pooling and incorporating the evidence from multiple studies to assess the potential impact of specific school dietary policies on CMD outcomes in the US.

### Policy implications

Evidence on the health impact of national policies targeting the school food environment is especially relevant and timely given the potentially evolving priorities of the new federal administration. Congress did not reauthorize the HHFKA as scheduled in Sept 2015, so the future of the Smart Snack Standards, which currently cover US students in 99% of public schools and 83% of private schools [46], remains uncertain. Our findings suggest health benefits of these standards during both childhood and later adulthood, supporting a need for reauthorization. Our results also suggest that most childhood SSB consumption will not be eliminated by school policies alone, making programmatic interventions to reduce SSB availability and intake in other venues important for children.

Currently, the national FFVP is available only for elementary schools with high proportions of low-income students [18], covering about 4 million students across 50 states and the territories [47]. Our investigation suggests meaningful health benefits of extending this program nationwide to elementary, middle, and high schools across the US. Indeed, our results indicate that the combination of F&V provision and SSB restriction has complementary health benefits.

### Strengths and limitations

Our analysis included nationally representative data on dietary intakes, BMI, demographics, and CMD mortality, increasing generalizability to the US population. Our estimates for the impact of these school policies on diet, as well as the etiologic effects of diet on CMD, were obtained from systematic reviews and meta-analyses, increasing validity of these estimates. Model inputs were stratified by age, sex, and race wherever relevant, accounting for differences in prevalent diets, BMI, and cardiometabolic mortality. We utilized conservative assumptions whenever possible, such as no significant effects of F&V intake on childhood BMI, reducing the likelihood we overestimated health benefits. We modeled and estimated effects in both children and adults, providing a clearer picture of overall population effects. Uncertainty was incorporated using Monte Carlo simulations; and sensitivity analyses tested the robustness of our results to changes in inputs and assumption.

Potential limitations should be considered. National estimates of dietary intakes were based on self-report, which could introduce error. To minimize this, we averaged two 24-hour recalls per person and accounted for within-person variation. The school interventions used to estimate the impact of these programs were of relatively brief duration (~1–2 years); and longer-term consecutive programs could have larger effects, particularly if there are lag-effects or acceleration of effects over time. The intervention studies varied in target populations, protocols, and implementation, and therefore are best considered as representing an average population effect of school policies for F&V provision and SSB restriction rather than being applied to any one child. For instance, additional technical assistance and resource support in implementing these policies could increase their impact. Although stochastic and parameter uncertainty were included in our UI estimates, we recognize the additional unquantifiable uncertainty, such as in the sustainability of these intervention effects. Direct evidence was not available on sustained impacts of these school policies on adult dietary habits, requiring estimation from other studies tracking diets from childhood into adulthood. For example, food habits from a school-based intervention may track differently into adulthood than a family-based change. To address this uncertainty, we conducted a sensitivity analysis on the sustained effects of these interventions on diet.

### Conclusions

These findings suggest that national school policies on F&V provision and SSB restriction in US elementary, middle, and high schools would modestly but meaningfully improve dietary habits and BMI in children and CMD mortality later in life. Our findings have implications for the public, school officials, health care providers, and policymakers aiming to improve the health and nutrition of children and reduce the burden of CMD in adults.

## Supporting information

S1 File Methods for estimating the relationship between SSB intake and BMI in children. (DOCX)

S2 File Comparative risk assessment modeling approach. (DOCX)

S1 Table Sources used to estimate the relationship between SSB intake and BMI in children. (DOCX)

S2 Table Model components, sources, and notes for analysis of impact of F&V provision and SSB restriction on child diet and BMI and future CMD outcomes. (DOCX)

S3 Table Select model inputs and estimated changes in child diet and child BMI associated with F&V provision and SSB restriction in US schools by child gender. (DOCX)

S4 Table Select model inputs and estimated changes in child diet and child BMI associated with F&V provision and SSB restriction in US schools by child race/ethnicity. (DOCX)

S5 Table Sensitivity analysis on change in BMI associated with F&V provision by child age. (DOCX)
