# Supplementary material for: Comparative risk assessment of school food environment policies and childhood diets, childhood obesity, and future cardiometabolic mortality in the United States
Source: PLoS One. 2018 Jul 6;13(7):e0200378. doi: 10.1371/journal.pone.0200378 (PMC6034872; doi:10.1371/journal.pone.0200378)
Supplement: S1 File — (DOCX) [file pone.0200378.s001.docx]

# S1 File. Methods for estimating the relationship between SSB Intake and BMI in children

We conducted a literature search for randomized controlled trials (RCTs), prospective cohort studies, and meta-analyses of these studies and identified three lines of evidence to quantify the relationship between changes in SSB intake and BMI in children (**Table S1**). Cross-sectional studies were not considered, nor were studies that only reported on binary changes in the risk or odds of overweight or obesity, rather than BMI. A meta-analysis by Malik et al. (2013) included 15 cohort studies in children and adolescents [1]. Many of these studies only evaluated baseline SSB intake, rather than change in SSB intake which has been shown to be more valid for estimating effects on BMI in observational studies [2]. Malik et al. (2013) also included a meta-analysis of 5 RCTs [1]. Of these trials, two RCTs were most appropriate for our analysis given their intervention modality (beverage substitution) and duration (12-18 months). These included RCTs led by de Ruyter et al. (2012) and Ebbeling et al. (2012) [3-4]. For each study, we extracted the findings as reported and also standardized to a standard (8 oz) SSB serving size and for BMI in kg/m^2^.

Per serving of SSBs, both RCTs provided relatively similar effect estimates on childhood BMI. Ebbeling et al. identified larger effects in overweight or obese children, compared with normal weight children. However, de Ruyter et al. identified a more conservative effect and also included a wider age range of youth, relevant to our investigation focused on children age 5-18 years. We therefore utilized this finding in our comparative risk assessment modeling. As previously recommended, we modeled effects of dietary changes on BMI in kg/m^2^ rather than BMI z-scores because within-child changes in BMI z-scores over time are contingent on adiposity status and BMI in kg/m^2^ is better for assessing longitudinal changes [5-6].

**References**

1. Malik VS, Pan A, Willett WC, Hu FB. Sugar-sweetened beverages and weight gain in children and adults: a systematic review and meta-analysis. *Am J Clin Nutr*. 2013;98(4):1084-1102.

2. Smith JD, Hou T, Hu FB, et al. A Comparison of Different Methods for Evaluating Diet, Physical Activity, and Long-Term Weight Gain in 3 Prospective Cohort Studies. *J Nutr*. 2015;145(11):2527-2534.

3. de Ruyter JC, Olthof MR, Seidell JC, Katan MB. A trial of sugar-free or sugar-sweetened beverages and body weight in children. *N Engl J Med*. 2012;367(15):1397-1406.

4. Ebbeling CB, Feldman HA, Chomitz VR, et al. A randomized trial of sugar-sweetened beverages and adolescent body weight. *N Engl J Med*. 2012;367(15):1407-1416.

5. Cole TJ, Faith MS, Pietrobelli A, Heo M. What is the best measure of adiposity change in growing children: BMI, BMI %, BMI z-score or BMI centile? *Eur J Clin Nutr*. 2005;59(3):419-425.

6. Berkey CS, Colditz GA. Adiposity in adolescents: change in actual BMI works better than change in BMI z score for longitudinal studies. *Ann Epidemiol*. 2007;17(1):44-50.
