# Supplementary material for: Comparative risk assessment of school food environment policies and childhood diets, childhood obesity, and future cardiometabolic mortality in the United States
Source: PLoS One. 2018 Jul 6;13(7):e0200378. doi: 10.1371/journal.pone.0200378 (PMC6034872; doi:10.1371/journal.pone.0200378)
Supplement: S2 File — (DOCX) [file pone.0200378.s002.docx]

# S2 File. Comparative risk assessment modeling approach

## Potential impact fraction

The standard description of the potential impact fraction (PIF) used to communicate that a comparative risk assessment framework has been used to estimate attributable mortality, gives only a vague insight as to how the PIF is calculated and what assumptions are being made. Because of this, we go into more detail here.

The PIF formula used is as follows:

$$\begin{matrix} \frac{\int_{x=0}^{m} RR(x)P(x)dx-\int_{x=0}^{m} RR'(x)P'(x)dx}{\int_{x=0}^{m} RR(x)P(x)dx} \end{matrix}$$

Where $P(x)$ is the distribution of current dietary consumption, ($P'(x)$) is the distribution of post-intervention dietary consumption, $RR(x)$ is the relative risk of mortality at exposure level $x$ pre-intervention, $RR'(x)$ is the relative risk of mortality at exposure level $x$ post-intervention and $m$ is the maximum exposure level.

## $\boldsymbol{P(x)}$ and $\boldsymbol{P'(x)}$

We assume current dietary intake follows a gamma distribution for all food and nutrient groups of interest. Previous research using comparative risk assessment in field of nutrition has assumed intake follows a normal distribution or some variation thereof. However, NHANES data show that intake is right skewed for the food and nutrient groups of interest (in some cases, as with nuts, extremely so), so we choose to assume intake follows a gamma distribution. Based on a visual inspection of histograms, we concluded that, overall, the gamma distribution fit the NHANES data better than an alternative right-skewed distribution (the log-normal), particularly for food groups where the intake is highly skewed, such as nuts. Simulations done to compare attributable mortality estimates assuming gamma, normal, and log-normal distributions to mortality estimates based on a non-parametric approach showed that estimates assuming the gamma distribution gave closer estimates to the non-parametric approach than the others.

Because the mean and variance of the gamma distribution is a function of the parameters of the gamma distribution ($E[X]=\frac{\alpha}{\beta}$, $Var[X]=\frac{\alpha}{\beta^{2}}$ where $X$ is a gamma random variable, $\alpha$ is the shape parameter and $\beta$ is the scale paraemter), estimates for the gamma parameter can be obtained from mean and variance estimates that account for survey design characteristics.

We assume post-intervention dietary consumption also follows a gamma distribution with mean of the current distribution plus the intervention effect, and standard deviation unchanged from the current distribution. Note that assuming each individual in the population experiences the same intervention effect would result in this post-intervention distribution.

##

$$\boldsymbol{RR(x)}$$

$RR(x)$ is defined to be

$$\begin{matrix} \left\{ \begin{matrix} exp(\beta(x-y(x))) & :x-y(x)\geq0 \\ 1 & :x-y(x)<0 \end{matrix} \right. \end{matrix}$$

where $\beta$ is the the change in log relative risk per unit of exposure, $x$ is the current exposure level, and $y(x)$ is the theoretical minimum risk exposure level. $y(x)$ is defined to be $F_{TMRED}(F_{X}^{-1}(x))$, where $F_{TMRED}$ is the cumulative distribution function of the theoretical minimum risk exposure distribution (TMRED) and $F_{X}^{-1}$ is the inverse cumulative distribution function of the current exposure distribution. Similarly, $RR'(x)$ is defined to be

$$\begin{matrix} \left\{ \begin{matrix} exp(\beta(x-y(x))) & :x-y'(x)\geq0 \\ 1 & :x-y'(x)<0 \end{matrix} \right. \end{matrix}$$

where $y(x)$ is defined to be $F_{TMRED}(F_{X}'^{-1}(x))$ and $F_{X}'^{-1}$ is the inverse cumulative distribution function of the counterfactual exposure.

Implicit in how we characterize the relative risk function are some of the fundamental assumptions we make about relative risk. Namely, that relative risk increases exponentially as distance from theoretical minimum risk exposure level ($y$) increases, that there is no risk attributable to exposure beyond the theoretical minimum risk exposure level, and that both $x$ and the theoretical minimum risk exposure level for an individual at exposure level $x$ are the $q$-th quantile of their respective distributions (the observed exposure distribution / counterfactual exposure distribution, and the TMRED, respectively). Note that, the change in relative risk per unit of exposure is assumed to be the same pre and post intervention. $RR(x)$ and $RR'(x)$ only differ because the theoretical minimum risk exposure level for an individual at exposure level $x$ differs.

##

$$\boldsymbol{m}$$

In our analyses, $m$ is defined to be $\infty$. Since the density of a gamma distribution approaches $0$ as exposure, $x$, approaches infinity, and because implausibly high values of exposure should exceed the corresponding theoretical maximum exposure level, implausibly high values of exposure will make no contributions to the PIF.

## Computation

In practice, we use simple numerical integration (using Riemann sums) to compute the integrals in the PIF formula. Thus, we use a categorical equivalent of the PIF formula

$$\begin{matrix} PIF=\frac{\sum_{i=1}^{n} P_{i}RR_{i}-\sum_{i'=1}^{n} P'_{i'}RR'_{'i}}{\sum_{i'=1}^{n} P_{i}RR_{i}} \end{matrix}$$

where the $n$ categories are determined by dividing up the exposure range (chosen here to be $\left[ 0,F_{X}^{-1}(\Phi(6)) \right]$ for current exposure and $\left[ 0,F_{X'}^{-1}(\Phi(6)) \right]$ for counterfactual exposure) into 121 intervals, each of length 0.1 when converted to the standard normal scale (except for the first one). More precisely, the range of exposure group $i$ can be described as follows:


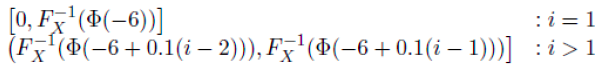


and for exposure group $i$’:


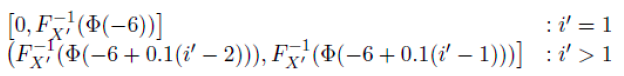


## Joint PIFs

Because summing would overestimate joint relationships [1], in instances where multiple dietary factors change in response to a policy or policies, we used the following formula to calculate joint population impact fractions (PIF_joint_) for each stratum and disease:

$${PIF}_{joint}=1- \prod_{r-1}^{R} (1-{PIF}_{r})$$

Where r denotes individual dietary exposures, and R is the number of dietary exposures. Joint distributions of exposures may be partially correlated among individuals, leading to overestimation of joint PIFs. However, validity analyses of dietary pattern studies in a separate publication showed that the estimated etiologic relationships of individual components and their joint associations were each reasonable [2].

## Monte Carlo Simulations

Monte Carlo simulations were used to quantity uncertainty in the PIFs, incorporating uncertainty of estimates of exposure means, etiologic RRs, and intervention effects. Specifically, for each diet disease pair and stratum, we drew randomly 1000 times from the normal distribution of the estimate of disease-specific change in the log(RR) corresponding to a one unit increase in intake, the normal distribution of the estimate of the exposure mean, and the normal distribution of the estimate of the intervention effect. Draws of mean intake that were zero or less were changed to 0.00001. Each set of random draws was was used to calculate the PIFs and attributable mortality.

## PIF via mediated effects

Consistent with the Global Burden of Disease Study (GBD) and evidence for harms of high body mass index (BMI) on specific cardiometabolic disease outcomes, the relationships of high intake of sugar‐sweetened beverages (SSBs) with coronary heart disease (CHD), hypertensive heart disease, ischemic stroke, hemorrhagic stroke (only when BMI ≥25 kg/m^2^), and diabetes were estimated through their measured relationships BMI (associations mediated by BMI) [3-4]. Direct relationships of SSBs with BMI [3-4], which are continuous changes in BMI rather than RRs, were also included. The association of change in BMI with change in SSB consumption was assessed using multivariate linear regression accounting for within‐person repeated measures, as described in earlier work [4-5]; separate linear relationships were estimated for BMI <25 and BMI ≥25 since the rate of associated increase in BMI due to SSB intake varies based on an individual's baseline BMI. Independently of this, additional direct relationships with CHD and diabetes (after adjustment for BMI) were included; total associations with diabetes (direct plus mediated; after excluding associations with diabetes mediated by BMI) were investigated in a previous publication [2]. We estimated log(RR) per unit associated increase in exposure for SSBs by taking the log(RR) per unit associated increase in exposure for BMI and multiplying it by an estimate of the associated increase in BMI per one unit associated increase in SSBs. For each stratum, the latter effect was weighted by the proportion of overweight (BMI ≥ 25 kg/m2) vs. non‐overweight (BMI < 25 kg/m2) individuals in that stratum, given the larger associations of SSBs with long‐term weight gain in overweight adults.

**References**

1. Lim SS, Vos T, Flaxman AD, et al. A comparative risk assessment of burden of disease and injury attributable to 67 risk factors and risk factor clusters in 21 regions, 1990-2010: a systematic analysis for the Global Burden of Disease Study 2010. *Lancet Lond Engl*. 2012;380(9859):2224-2260. doi:10.1016/S0140-6736(12)61766-8.

2. Micha R, Peñalvo JL, Cudhea F, Imamura F, Rehm CD, Mozaffarian D. Association Between Dietary Factors and Mortality From Heart Disease, Stroke, and Type 2 Diabetes in the United States. *JAMA*. 2017;317(9):912-924. doi:10.1001/jama.2017.0947.

3. Singh GM, Danaei G, Pelizzari PM, et al. The age associations of blood pressure, cholesterol, and glucose: analysis of health examination surveys from international populations. *Circulation*. 2012;125(18):2204-2211. doi:10.1161/CIRCULATIONAHA.111.058834.

4. Singh GM, Micha R, Khatibzadeh S, et al. Estimated Global, Regional, and National Disease Burdens Related to Sugar-Sweetened Beverage Consumption in 2010. *Circulation*. 2015;132(8):639-666. doi:10.1161/CIRCULATIONAHA.114.010636.

5. Mozaffarian D, Hao T, Rimm EB, Willett WC, Hu FB. Changes in Diet and Lifestyle and Long-Term Weight Gain in Women and Men. *N Engl J Med*. 2011;364(25):2392-2404. doi:10.1056/NEJMoa1014296.
