# Supplementary material for: Comparative risk assessment of school food environment policies and childhood diets, childhood obesity, and future cardiometabolic mortality in the United States
Source: PLoS One. 2018 Jul 6;13(7):e0200378. doi: 10.1371/journal.pone.0200378 (PMC6034872; doi:10.1371/journal.pone.0200378)
Supplement: S1 Table — (DOCX) [file pone.0200378.s003.docx]

**S1 Table. Sources used to estimate the relationship between SSB intake and BMI in children.**

| **Source** | **Study design** | **Outcome** | **Unstandardized effect size^d^** | **Standardized effect size^e^** |
| --- | --- | --- | --- | --- |
| de Ruyter et al. 2012^a^ | RCT | Mean difference in change in BMI z-score over study duration (control-intervention) | -0.13 SD units  (SE=-0.04) | 0.43 kg/m^2^ (SE=0.06)^f^ |
| Ebbeling et al. 2012^b^ | RCT | Mean difference in change in BMI over study duration (control-intervention) | -0.57 kg/m^2^ (SE=0.28) | 0.54 kg/m^2^ (SE =0.01) |
| Malik et al. 2013^c^ | Meta-analysis | Change in BMI and BMI z-score | 0.07 kg/m^2^ (SE=0.03) | 0.05 kg/m^2^ (SE=0.02)^h^ |
| ^a^ This study assessed the effect of replacing SSBs with non-caloric beverages on BMI. A total of 641 normal weight children were included, ranging from 4.8-11.9 years of age, and the study duration was 18 months. | | | | |
| ^b^ This study assessed the effect of replacing SSBs with non-caloric beverages on BMI z-score, and the study duration was one year. The study included 224 overweight and obese adolescents in grades 9-10 (mean age at baseline = 15.3y) who consume at least one 12 oz serving of SSBs per day. While one- and two-year effects were reported, we only included the one-year effect because the intervention was one year in duration. | | | | |
| ^c^ Reported results are for meta-analysis including 15 cohort studies with sample sizes ranging from 141-11703 children and adolescents ages 2-16 years at baseline. Study durations ranged from 6 months to 14 years. | | | | |
| ^d^ Unstandardized effect sizes are the effect sizes as reported in each publication. | | | | |
| ^e^ Standardized to 8oz serving size after incorporating estimates of adherence, when possible. | | | | |
| ^f^ The LMS equation (http://www.cdc.gov/growthcharts/percentile_data_files.htm)  was used to convert the results from de Ruyter into changes in absolute BMI. In addition, the authors reported 83% adherence, which corresponds to a serving size of 6.64 oz. The effect size was re-scaled from a 6.64 oz serving to an 8 oz serving. | | | | |
| ^g^ The authors reported a 0.7 serving/day difference in SSB intake between the intervention and control group, which corresponds to on observed intake of 8.4 oz. serving/day (or 70% adherence). The effect size was re-scaled to correspond to a change in SSB intake of 8oz/day. | | | | |
| ^h^ Converted from 12 oz to 8 oz per day serving of SSBs. | | | | |
