# Supplementary material for: Comparative risk assessment of school food environment policies and childhood diets, childhood obesity, and future cardiometabolic mortality in the United States
Source: PLoS One. 2018 Jul 6;13(7):e0200378. doi: 10.1371/journal.pone.0200378 (PMC6034872; doi:10.1371/journal.pone.0200378)
Supplement: S2 Table — (DOCX) [file pone.0200378.s004.docx]

**S2 Table. Model components, sources, and notes for analysis of impact of F&V provision and SSB restriction on child diet and BMI and future CMD outcomes.**

| **Model component** | | **Source** | **Notes** |
| --- | --- | --- | --- |
| ***Change in dietary intake and change in BMI in children*** | | | |
| **Demographics** | |  |  |
|  | Age, gender, race/ethnicity | NHANES 2009-10 and 2011-12, ages 5-18 | We included demographic data from all children from the two most recent cycles of NHANES under the assumption that US children in elementary, middle, and high school would be subject to F&V provision and SSB restriction. Students attending private schools were not excluded because they constitute less than 10% of all US students and because the majority of private schools are tax-exempt and non-profit, which could make them subject to national school food environment policies. School levels were defined by age such that 5-10 year-olds were labeled as elementary school students, 11-14 year-olds were labeled as middle school students, and 14-18 year-olds were labeled as high school students. Racial/ethnic groups included non-Hispanic white, non-Hispanic black, Hispanic, and other. |
| **Baseline characteristics** | |  |  |
|  | Dietary intake, BMI | NHANES 2009-10 and 2011-12, ages 5-18 stratified by age, race/ethnicity, and sex | We included dietary intake and BMI data from all children from the two most recent cycles in NHANES under the assumption that US children in elementary, middle, and high school would be subject to F&V provision and SSB restriction (see explanation above). We accounted for survey design and sample weights. For dietary intake data, we combined data from two nonconsecutive 24-hour dietary recalls. Demographic data from NHANES were used to stratify dietary intake and BMI data by age, race/ethnicity, and sex. |
| **Effect of F&V provision and SSB restriction on diet** | | Micha et al., 2017a^1^ | Estimates of the impact of F&V provision and SSB restriction on absolute change in dietary intake were obtained from a meta-analysis including 15 trials on F&V provision and 3 trials on SSB restriction. This demonstrated an average 0.27 serving/day (95% CI: 0.17, 0.36) increase in fruits (80g/serving), 0.04 servings/day (95% CI: 0.01, 0.08) increase in vegetables (80g/serving), and 0.18 servings/day (95% CI: -0.47, -0.07) decrease in SSBs (8oz/serving). This meta-analysis included F&V provision and SSB restriction interventions lasting approximately 1-2 years; therefore, our estimates for changes in diet and BMI in children reflect short-term effects of such policies. |
| **S2 Table. (continued)** | | | |
| **Model component** | | **Source** | **Notes** |
| ***Change in dietary intake and change in BMI in children*** | | | |
| **Effect of F&V provision and SSB restriction on BMI** | |  |  |
|  | F&V provision | Personal communication, D Mozaffarian, Dec 13 2016^2^ | Due to insufficient evidence linking F&V intake to BMI in childhood, we conservatively assumed no effects of F&V provision on childhood BMI in our main analysis. In a sensitivity analysis, we estimated the effects of changes in F&V intake on BMI from an unpublished analysis of three prospective cohort studies in adults (Nurses Health Study, Nurses Health Study2, and Health Professionals Follow-Up Study). Point estimates and 95% uncertainty intervals for the changes in BMI associated with changes in F&V intake were derived from probabilistic sensitivity analysis sampling from the distribution of baseline BMI (mean, SE) from NHANES, the estimated effects of F&V provision on F&V intake from a meta-analysis of school food environment interventions, and an estimate of the relationship between changes in F&V intake from the aforementioned cohort studies in adults. |
|  | SSB restriction | de Ruyter et al., 2012^3^ | The effect of change in SSB intake on BMI was derived from a randomized controlled trial. Point estimates and 95% uncertainty intervals for the changes in BMI associated with changes in SSB intake were derived from probabilistic sensitivity analysis sampling from the distribution of baseline BMI (mean, SE) from NHANES, the estimated effects of SSB restriction on SSB intake from a meta-analysis of school food environment interventions, and an estimate of the relationship between changes in SSB intake and BMI from a RCT. |
| ***Change in CMD mortality in adults*** | | |  |
| **Demographics** | |  |  |
|  | Age, race/ethnicity, gender | NHANES 2009-10 and 2011-12, ages 25+ | Age categories included: 25-34, 35-44, 45-54, 55-64, 65-74, 75+ years. Race/ethnicity categories included non-Hispanic white, non-Hispanic black, Hispanic, and other. |
| **Baseline characteristics** | |  |  |
|  | Dietary intake, BMI | NHANES 2009-10 and 2011-12, ages 25+ stratified by age, race/ethnicity, and sex | Dietary data were averaged from two 24-hour recalls. For dietary intake and BMI data, we used survey weights that accounted for the sample design and probabilities of selection. Demographic data from NHANES were used to stratify dietary intake and BMI data by age, race/ethnicity, and sex. |
| **S2 Table. (continued)** | | | |
| **Model component** | | **Source** | **Notes** |
| ***Change in CMD mortality in adults*** | | | |
| **Effect of F&V provision and SSB restriction on diet** | |  |  |
|  | Effect in childhood | Micha et al., 2017a^1^ | Effects of F&V provision and SSB restriction in childhood were obtained from a meta-analysis of school food environment interventions. Because these interventions were only approximately 1-2 years in duration, which is much shorter than typical school food environment policies, we assumed that the effects of these policies on diet would be additive across each level of schooling (elementary, middle, and high school) if the policies had been in place when current US adults were children. |
|  | Effect sustained to adulthood | Craigie et al., 2011^4^ | We modeled 35% of dietary changes in childhood associated with these policies being sustained into adulthood, based on evidence in a systematic review on within-individual correlations of dietary habits in childhood and adulthood. In sensitivity analyses, we considered smaller (25%) and larger (50%) sustained changes in adulthood. |
| **Etiologic effects of dietary changes on disease outcomes** | | Micha et al., 2017b^5^ | RR estimates correspond to a one serving increase in dietary intake for all adults, stratified by age, with additional stratifications by race/ethnicity and sex when appropriate. RR estimates were obtained from meta-analyses of cohort studies, with updated RRs and 95% confidence intervals from our work in the 2010 Global Burden of Diseases Study. There were no differential effects on CMD incidence versus CMD cause-specific mortality; therefore, mortality effects were assumed to similar to effects on incidence. |
| **Baseline mortality estimates (CHD, diabetes, stroke)** | | National Center for Health Statistics’ Division of Vital Statistics 2012 | Current mortality estimates for stroke, CHD, and type 2 diabetes were stratified by age, sex, and race/ethnicity. We included deaths from the following diseases: CHD (ICD 10: I20-I25), ischemic stroke (I63, I65-I67 [except I67.4], I69.3, G45), hemorrhagic stroke (I60-I62, I69.0-I69.2, I67.4), unidentified and other non-ischemic/hemorrhagic stroke (I64, I69.4, I69.8), and type 2 diabetes mellitus (E10-E14 [except E10.2, E11.2, E12.2, E13.2]). |
| Abbreviations: BMI, body mass index; NHANES, National Health and Nutrition Examination Survey; F&V, fruit and vegetable; SSB, sugar-sweetened beverage; CMD, cardiometabolic disease; RCT, randomized controlled trial; CHD, coronary heart disease; RR, risk ratio | | | |
| ^1^ Micha R, Karageorgou D, Bakogianni I, et al. Effectiveness of School Food Policies on Children’s Dietary Behaviors: A Systematic Review and Meta-Analysis. Submitted | | | |
| ^2^ The effects of changes in F&V intake on BMI were derived from unpublished results from an analysis of three prospective cohort studies in adults (Nurses Health Study, Nurses Health Study2, Health Professionals Follow-Up Study). Results obtained through personal communication with Mozaffarian, D, Dec 13, 2016. | | | |
| ^3^de Ruyter JC, Olthof MR, Seidell JC, Katan MB. A trial of sugar-free or sugar-sweetened beverages and body weight in children. *N Engl J Med*. 2012;367(15):1397-1406. | | | |
| ^4^Craigie AM, Lake AA, Kelly SA, Adamson AJ, Mathers JC. Tracking of obesity-related behaviours from childhood to adulthood: A systematic review. *Maturitas*. 2011;70(3):266-284. | | | |
| ^5^ Micha R, Peñalvo JL, Cudhea F, Imamura F, Rehm CD, Mozaffarian D. Association Between Dietary Factors and Mortality From Heart Disease, Stroke, and Type 2 Diabetes in the United States. JAMA 2017;317(9):912–24. | | | |
