# Supplementary material for: Comparative risk assessment of school food environment policies and childhood diets, childhood obesity, and future cardiometabolic mortality in the United States
Source: PLoS One. 2018 Jul 6;13(7):e0200378. doi: 10.1371/journal.pone.0200378 (PMC6034872; doi:10.1371/journal.pone.0200378)
Supplement: S4 Table — (DOCX) [file pone.0200378.s006.docx]

**S4 Table. Select model inputs and estimated changes in child diet and child BMI associated with F&V provision and SSB restriction in US schools by child race/ethnicity.^1^**

| **Dietary exposure** | **Child race/**  **ethnicity** | **Baseline dietary intake, mean servings/d (SE)^2^** | **Dietary change associated with F&V provision and SSB restriction (servings/d)** | | **Baseline BMI (kg/m^2^), mean (SE)^5^** | **BMI change associated with SSB restriction^5^** | |
| --- | --- | --- | --- | --- | --- | --- | --- |
|  |  |  | **Mean (SE)^3^** | **% (95% UI)^4^** |  | **Median**  **(95% UI)** | **%**  **(95% UI)** |
| **Fruits** (80g/svg) |  |  |  |  |  |  |  |
|  | NHW | 1.33 (0.11) | 0.27 (0.05) | 19.9  (12.4, 28.4) | 20.6 (0.2) | NA | NA |
|  | NHB | 0.97 (0.04) | 0.27 (0.05) | 27.4  (17.3, 37.6) | 22.2 (0.3) | NA | NA |
|  | HISP | 1.47 (0.07) | 0.27 (0.05) | 18.1  (11.4, 25.1) | 21.4 (0.1) | NA | NA |
|  | OTH | 1.27 (0.13) | 0.27 (0.05) | 21.0  (13.0, 30.9) | 20.5 (0.4) | NA | NA |
| **Vegetables** (80g/svg) |  |  |  |  |  |  |  |
|  | NHW | 1.14 (0.06) | 0.04 (0.02) | 3.9  (0.4, 7.4) | 20.6 (0.2) | NA | NA |
|  | NHB | 1.07 (0.06) | 0.04 (0.02) | 4.1  (0.4, 7.9) | 22.2 (0.3) | NA | NA |
|  | HISP | 1.48 (0.04) | 0.04 (0.02) | 3.0  (0.4, 5.7) | 21.4 (0.1) | NA | NA |
|  | OTH | 1.48 (0.14) | 0.04 (0.02) | 3.0  (0.3, 5.8) | 20.5 (0.4) | NA | NA |
| **SSBs** (8oz/svg) |  |  |  |  |  |  |  |
|  | NHW | 1.45 (0.06) | -0.27 (0.10) | -18.8  (-32.7, -4.5) | 20.6 (0.2) | -0.1  (-0.2, -0.03) | -0.6  (-1.0, -0.1) |
|  | NHB | 1.46 (0.07) | -0.27 (0.10) | -18.8  (-33.0, -4.4) | 22.2 (0.3) | -0.1  (-0.2, -0.03) | -0.5  (-1.0, -0.2) |
|  | HISP | 1.31 (0.06) | -0.27 (0.10) | -20.6  (-36.0, -5.5) | 21.4 (0.1) | -0.1  (-0.2, -0.03) | -0.6  (-1.0, -0.2) |
|  | OTH | 1.00 (0.07) | -0.27 (0.10) | -26.8  (-48.5, -6.3) | 20.5 (0.4) | -0.1  (-0.2, -0.03) | -0.6  (-1.0, -0.1) |

Abbreviations: SSBs, sugar-sweetened beverage; F&V, fruit and vegetable; NHW, non-Hispanic White; NHB; non-Hispanic Black; HISP, Hispanic; OTH, other; SE, standard error; UI, uncertainty interval; BMI, body mass index; NA, not applicable.

^1^ Outcomes were modeled assuming all US children in elementary, middle, and high school would be subject to F&V provision and SSB restriction. Students in private schools were not excluded because they constitute less than 10% of all US students and because the majority of private schools (tax-exempt, non-profit) could be subject to these policies. Outcomes are estimated using inputs for the effects of short-term (1-2 years) school food environment interventions on diet; no assumptions are made about the potential effects of longer-term policies on child diet and BMI.

^2^ Included the two most recent cycles of the National Health and Nutrition Examination Survey (NHANES; 2009-10 and 2011-12); N=4,165 children ages 5-18 yrs. We accounted for survey design and sample weights, and averaged data from two nonconsecutive 24-hour dietary recalls. Energy-adjusted dietary intakes were calculated using the residual method.

^3^ Estimates of the impact of F&V provision and SSB restriction on absolute change (mean, SE) in dietary intake were obtained from a meta-analysis including 18 school food environment intervention studies. Studies included in this meta-analysis include interventions with durations ranging from approximately 1-2 years; therefore, estimated results for diet and BMI reflect the effects of these short-term interventions on absolute change in intake.

^4^ Point estimates and 95% uncertainty intervals for the percent change in dietary intake associated with F&V provision and SSB restriction were obtained from a probabilistic sensitivity analysis sampling from the distribution of dietary intakes of fruits, vegetables, and SSBs (mean, SE) obtained NHANES and the estimated effect of these policies on diet from a meta-analysis of school food environment intervention studies. The percent change is the median estimate from 1000 Monte Carlo simulations and the 95% uncertainty intervals are the 2.5^th^ and 97^th^ percentiles of the percent change.

^5^ Baseline (without policy) BMI data were obtained from the two most recent cycles of NHANES (2009-10 and 2011-12); N=4723. The effect of changes in SSB intake on BMI was derived from a randomized controlled trial. Point estimates and 95% uncertainty intervals for the absolute and percent change in BMI were derived from probabilistic sensitivity analysis sampling from the distribution of baseline BMI (mean, SE) from NHANES, the estimated effects of SSB restrictions on SSB intake from a meta-analysis of school food environment interventions, and estimates for the relationships between changes in SSB intake and BMI from an RCT. The point estimates (median and percent change) are the median estimates from 1000 Monte Carlo simulations and the 95% uncertainty intervals are the 2.5^th^ and 97^th^ percentiles of the absolute and percent change in BMI. Due to insufficient evidence linking fruit and vegetable intake to BMI in childhood, we conservatively assumed no effects of F&V changes on childhood BMI in our main analysis.
