# Supplementary material for: Comparative risk assessment of school food environment policies and childhood diets, childhood obesity, and future cardiometabolic mortality in the United States
Source: PLoS One. 2018 Jul 6;13(7):e0200378. doi: 10.1371/journal.pone.0200378 (PMC6034872; doi:10.1371/journal.pone.0200378)
Supplement: S5 Table — (DOCX) [file pone.0200378.s007.docx]

**S5 Table. Sensitivity analysis on change in BMI associated with F&V provision by child age.**

| **Dietary exposure** | **Age Group** | **Estimated change in BMI per serving/day increase in F&V**  **(mean, SE)^1^** | **BMI (kg/m^2^) without F&V provision, mean (95% CI)^2^** | **BMI change associated with F&V provision^3^** | |  |
| --- | --- | --- | --- | --- | --- | --- |
|  |  |  |  | **Median (95% UI)** | **% (95% UI)** |  |
| **Fruit** (80g/svg) | Elementary school | -0.06 (0.02) | 17.8 (17.6, 17.9) | -0.02 (-0.03, -0.01) | -0.09 (-0.15, -0.05) |  |
|  | Middle school | -0.06 (0.02) | 22.0 (21.6, 22.5) | -0.02 (-0.03, -0.01) | -0.07 (-0.12, -0.04) |  |
|  | High school | -0.06 (0.02) | 24.6 (24.1, 25.0) | -0.02 (-0.03, -0.01) | -0.07 (-0.11, -0.04) |  |
| **Vegetables** (80g/svg) | Elementary school | -0.03 (0.01) | 17.8 (17.6, 17.9) | 0.00 (0.00, 0.00) | 0.01 (-0.02, 0.00) |  |
|  | Middle school | -0.03 (0.01) | 22.0 (21.6, 22.5) | 0.00 (0.00, 0.00) | 0.01 (-0.02, 0.00) |  |
|  | High school | -0.03 (0.01) | 24.6 (24.1, 25.0) | 0.00 (0.00, 0.00) | 0.00 (-0.01, 0.00) |  |
| Abbreviations: F&V, fruit and vegetable; BMI, body mass index; CI, confidence interval; UI, uncertainty interval | | | | | | |
| ^1^ The effects of changes in F&V intake on BMI were derived from an analysis of three prospective cohort studies in adults (Nurses Health Study, Nurses Health Study2, Health Professionals Follow-Up Study). | | | | | | |
| ^2^ Baseline (without policy) BMI data were obtained from the two most recent cycles of NHANES (2009-10 and 2011-12); N=4723. | | | | | | |
| ^3^ Point estimates and 95% uncertainty intervals were derived from probabilistic sensitivity analysis sampling from the distribution of baseline BMI (mean, SE) from NHANES, the estimated effects of these policies on dietary change from a meta-analysis of school food environment interventions, and estimates for the relationships between changes in dietary intake and BMI from cohort studies. The estimated median BMI and percent change in BMI associated with F&V provision are the median estimates from 1000 Monte Carlo simulations and the 95% uncertainty intervals are the 2.5^th^ and 97^th^ percentiles of the estimates. | | | | | | |
